# Sentiment analysis for cruises in Saudi Arabia on social media platforms using machine learning algorithms

**DOI:** 10.1186/s40537-022-00568-5

**Published:** 2022-02-18

**Authors:** Bador Al sari, Rawan Alkhaldi, Dalia Alsaffar, Tahani Alkhaldi, Hanan Almaymuni, Norah Alnaim, Najwa Alghamdi, Sunday O. Olatunji

**Affiliations:** 1 Department of Computer Science, Imam Abdulrahman Bin Faisal University, Dammam, Saudi Arabia; 2 Department of Information Technology, King Saud University, Riyadh, Saudi Arabia; 3 College of Computer Science and Information Technology, Imam Abdulrahman Bin Faisal University, Dammam, Saudi Arabia

**Keywords:** Sentiment analysis, Social media, Machine learning, Artificial intelligence, Cruise, Tourism

## Abstract

Social media﻿ has great importance in the community for discussing many events and sharing them with others. The primary goal of this research is to study the quality of the sentiment analysis (SA) of impressions about Saudi cruises, as a first event, by creating datasets from three selected social media platforms (Instagram, Snapchat, and Twitter). The outcome of this study will help in understanding opinions of passengers and viewers about their first Saudi cruise experiences by analyzing their feelings from social media posts. After cleaning, this experiment contains 1200 samples. The data was classified into positive or negative classes using the choice of machine learning algorithms, such as multilayer perceptron (MLP), naıve bayes (NB), random forest (RF), support vector machine (SVM), and voting. The results show the highest classification accuracy for the RF algorithm, as it achieved 100% accuracy with over-sampled data from Snapchat using both test options. The algorithms were compared among the three different datasets. All algorithms achieved a high level of accuracy. Hence, the results show that 80% of the sentiments were positive while 20% were negative.

## Introduction

Social media has undergone significant development in recent years; thus, a huge amount of information is in circulation. Various websites have been developed through which users can express their opinions and share their content. This is especially the case with the expansion of social networks (blogs, forums, and social media) in which the content is usually subjective and loaded with opinions and ratings. This kind of information can be very useful for recommending products or brands [1]. First, there is Twitter, which is a microblogging service that allows small blog posts called Tweets to be sent and received [2]. Second, Snapchat is a mobile messaging app for sharing temporary photos and videos called Snaps that disappear after viewing [3]. One of Snapchat’s most important features is the Snap Map that displays a real-time location for anyone who submits a snap to the map. The third platform is Instagram, which is commonly used to post photos and videos in order to share them with followers who can comment on or ‘like’ these posts. Social media provides an enormous amount of data. As a result, there is a need for data mining, which enables analysis of social media data and user sentiments by seeking their opinions on specific topics Saudi Arabia presented plans to change the course of its tourism sector through the development of Vision 2030 that was announced on 25 April 2016 by Crown Prince Mohammad bin Salman [4]. One of these plans is to invest in tourism by launching various events to attract visitors. Some of these events are unprecedented for the Saudi population, as they have been offered for the first time. For the first time, by offering tourist visas, Saudi Arabia was opening its doors to visitors from many countries. Saudi Arabia received 24,000 international visitors during the first ten days of applications for immediate tourist visas [5]. This study aims to analyze passengers’ and viewers’ opinions to see if the pandemic effects on the economy about cruise entertainment, which is the first of its kind in the Kingdom of Saudi Arabia [6, 7]. The sentiment analysis (SA) process is the systematic identification, extraction and quantification of affective states and subjective information using natural language processing [8]. It was made by starting with the collection of opinions as textual data from several social media platforms. The platforms used are Instagram, Snapchat and Twitter, because of their popularity in Saudi Arabia [9].

The opinions of this Red Sea Saudi cruise were analyzed and classified into negative and positive classes. To the best of our knowledge, this research is one of the few studies that classifies emotions by applying machine learning (ML) algorithms to Arabic datasets. This is because of the difficulty of finding logical results and the need for longer pre-processing steps. Furthermore, this study was launched during the Covid-19 pandemic. We study the quality of the sentiment analysis by various ML algorithms for the three selected social media platforms. Five of the most popular ML algorithms were applied: multilayer perceptron (MLP), Naıve Bayes (NB), random forest (RF), support vector machine (SVM), and the voting ensemble algorithm. These algorithms were used to classify opinions about the cruise. Each algorithm relies on a unique method for making predictions. Likewise, ML algorithms were chosen due to the size of the dataset. Finally, a comparison is made to evaluate the efficiency of these models in classifying textual data in the Arabic language.

The remaining of this paper is divided into five sections: “Literature review” section covers related work on SA in tourism. Next, the proposed techniques in this paper are presented, followed by the empirical and experimental studies, after which the results are discussed. Finally, the conclusion is presented along with ideas for potential for future work.

## Literature review

In this section, a literature review of the relevant research is provided. The research is summarized and classified based on the platform type used.

### Instagram

In  [10], the authors searched for a study of criteria for expressing feelings on social media, especially on Facebook, Twitter, Instagram and WhatsApp, and compared their efficacy for expressing six separate feelings. Through the analysis of the samples and the procedures, the results for expressing negative feelings show WhatsApp to be most suitable, followed by Facebook, Twitter, and Instagram. In order to ex- press positive feelings, perceived suitability was highest for WhatsApp, followed by Instagram, Facebook and Twitter. The system only provides a comparative analysis among these four platforms. In another study [11], the authors addressed the problem of predicting the success of music albums by investigating various data sources from social media to mainstream American newspapers. The principal technique applied was the RF approach, which predicted results with an accuracy of 94%. There are limitations regarding the shortness of the data collection period, which is only one month. In [12], the authors explored the use of Instagram to promote tourism destinations in Indonesia. By exploring users’ perceptions using in-depth conversations and interviews with visual styles and image-induction techniques, they tried to describe the potential value of Instagram for promoting tourism sites in Indonesia. They found everyone tried to promote their own cities in their own ways, with Instagram providing complete communication facilities from tourism brands to allow user-generated photographic content. The search was limited only to the Instagram platform’s contribution to the development of tourist destinations.

### Snapchat

In recent research [13], the authors investigated data posted to our story on the Snap Map. They collected photos and videos, and applied statistical and deep learning techniques to SA. The data were gathered during three events in Riyadh Tourist Season. Their results indicated the capacity for SA through Snapchat. The authors of [14] analyzed combined data from a questionnaire, Snapchat, and Google Maps. They looked into lexicon-based and ML approaches. The research results revealed that celebrities on Snapchat impact people’s choices of restaurants. In [3], the authors researched how US media uses Snapchat to reach young audiences. The chief technique applied was interviews and content analysis. The principal result showed that publishers on Snapchat Discover are embracing the capabilities of Snapchat, and adapting media types and story themes using visuals. Results also showed that the media retains its own character in judging the news. The system only dealt with the use of Snapchat Discover. Piwek and Joinson [15] ran an online survey using the memory sampling method to inquire into details of a recent photo sent by every Snapchat participant. Results showed that they already share ‘avatars’ and ‘creative logo graphics’, and often use them at home mainly as an easier and more fun way of reaching friends. In [8], the authors performed SA on social media textual data as a rich source of opinions. These textual views were classified into four categories based on their level of extremeness: low, high, moderate and neutral. To classify the data, multinomial NB and linear SVM classifier algorithms were used. The results showed that the SVM algorithm was the most accurate classifier with an accuracy of 82%.

### Twitter

In [16], the authors analyzed tweets collected in the Arabic language and compared different algorithms using SA with different n-grams as a method for feature extraction. The performance of the algorithms was evaluated by measuring accuracy, precision, recall, and f-measure. The result showed a 99.96% accuracy with unigram.

Also, Heikal et al. [17] explored a deep learning model for application to Arabic data in order to improve the accuracy of Arabic SA. The fundamental techniques were CNN and long-term memory models. The major result of this study was that the model achieved an F1 score of 64.46%, which outperformed the modern deep learning model’s F1 score of 53.6% for the Arabic sentiment dataset. The system was limited to analyzing sentiments from Twitter data only. In [18], the authors conducted an SA of social media. They applied the NB method and Google Prediction API. The accuracy achieved and the macro-F-measure were 90.21% and 89.98%, respectively. The main finding evaluated the classification performance by comparing it with predictions of the winner of the 2016 US election. However, only Twitter data were used. Furthermore, the authors looked at and discussed social media analysis using Twitter data relating to cruises, representing it in three categories of user group: commercial, news/blogs, and private [19]. Block analysis was the key method used after using three distinct techniques: word repetition, content analysis, and network analysis. Results showed tourists are less influential than celebrities, and celebrity influence is one of the marketing strategies that is relied upon nowadays. The data collection period was short, and sadly, the analysis remains mainly exploratory for this reason. In [20], the authors proposed hybrid algorithms to discover people’s opinions from their Twitter posts. The primary technique, the polarity classification algorithm, contained three stages for classifying 2,116 tweets into positive, negative, or natural groups. The central finding was that this achieved a greater accuracy than other algorithms for the same dataset. The paper evaluated the algorithm by using different metrics, although the authors did not indicate the keywords or the data collection period. In [21], the authors conducted SA of tweets to understand of the effect of the COVID-19 pandemic on the cruise industry, and mined semantic time-series data from social media. They computed the adjusted sentiment score for each tweet posted between 1 February and 18 June 2020. The main finding was that there are two groups, with the first suffering from quarantine and limits on travel because of COVID-19, making them even more eager to travel and explore, and the second, interested in cruise tourism possibly shifting from mass cruises to niche cruises.

### Other social networks

In a recent paper [22], the authors analyzed reviews on the TripAdvisor website. They applied multi-classification to get high performance of the SVM algorithm, NB over-sampling, Word2vec, and Knowledge Graph. The best result achieved was a recall of 0.901. As for places, the Tower of London was the best. Banati et al., [23] analyzed the emotions expressed by users about their experiences while traveling. Opinion mining was applied to reviews from the TripAdvisor website which were extracted using a web crawler in Python. The extracted reviews were classified as positive or negative at different levels: document level, sentence level, and feature/entity level. Classification for multiple entities at the document level could not be linked under the same category. In addition, they evaluated the performance of seven ML algorithms, such as RF, RT, NB, and OneR. The best accuracy achieved was for RF at 88.25%, while OneR provided the lowest result, with an accuracy of 68.1%. In addition [24], the authors considered the problem of the glut of information on the Internet discovered while mining reviews from travel blogs. They applied NB and SVM, with the main finding being that the SVM model with N- gram achieved excellent results. However, the system only dealt with the use of sentiment classification for reviews.

Brida et al. [25] considered the experiences of passengers on cruises and their features. The main technique applied was a decision tree (DT), with the authors analyzing data from 1361 responses collected through a questionnaire over three months in 2009. The main finding for the applied DT was an accuracy of 67.6%. However, the authors observed that the lower the characteristics were, the more accurate was the prediction. The paper does not consider different types of evalu-ation nor comparison of algorithms. In study [26], the authors focused on the SA of multilingual textual data from social media to discover the intensity of the sentiments for extremism. They proposed a manual method that effectively found extreme sentiment from multilingual data by creating a new multilingual lexicon or dictionary. Experiments were performed for supervised and unsupervised algorithms. The greatest accuracy achieved for SVM supervised was 82%, while for KNN unsupervised, the best accuracy was 26%.

In [27], the authors presented a data-driven approach to analyze data about trips from location-based social networks (LBSN). The study aimed to discover the mobility pattern for how tourists would travel the world. Moreover, they presented two applications to use the data from each trip. First, travelers were clustered in terms of the Twitter and Foursquare datasets, which obtained three clusters for Twitter and six for Foursquare. The second application area was the spatial clustering of destinations throughout the world. They identified 942 regions as destinations that can be directly used in a regional model for a destination recommender system. However, the results might have been affected by travelers’ continuously location- sharing their LBSNs, resulting in out-of-date datasets from Foursquare and Flickr. Table 1 shows a summary of the relevant studies. It is clear from the literature review that there are many studies in the SA field that have reported useful results. Nevertheless, the literature lacks comparative studies that use different social media platforms to analyze tourist impressions of new tourism events. A comparison of the performance of ML algorithms is made among several popular algorithms, such as MLP, SVM, RF, NB, and Voting based upon their accuracy rates. The experiment is tested using 10-fold cross-validation with the 70% split test option.

**Table 1:** Summary of previous studies

References	Data source	Method	Domain	Language	Size of data
[10]	Instagram, Twitter, Facebook, WhatsApp	Analysis and procedure	Emotion	Dutch	1201
[11]	Instagram, Twitter, Facebook	RF	Music	English	86 albums
[12]	Instagram	Dialogues, interviews	Tourists	English	–
[13]	Snapchat	Deep learning	Tourists	Arabic	–
[14]	Snapchat	Lexicon, ML	Economic	Arabic	1435 restaurant-goers
[3]	Snapchat	Content, Interviews	News	English	726 snaps
[15]	Snapchat	memory sampling	SA	English	online survey
[8]	Snapchat	NB, SVM	SA	English	textual data
[16]	Twitter	ML	SA	Arabic	151,500 tweets
[17]	Twitter	CNN, LSTM	SA	Arabic	–
[18]	Twitter	NB, Google prediction API	SA	English	120,000 tweets
[19]	Twitter	Word frequency	Tourists	English	42,785 tweets
[20]	Twitter	PCA	SA	English	2116 tweets
[21]	Twitter	lexicons	Tourists	English	53,546 tweets
[22]	TripAdvisor	SVM	Tourists	English	Reviews
[23]	TripAdvisor	ML	Tourists	English	2116 tweets
[24]	Online reviews	ML	Tourists	English	–
[25]	Questionnaire	DT	Tourists	English	1361 responses
[26]	Social media	NB, SVM	SA	English	2500 web pages
[27]	Social media	ML	Tourists	English	942 Regions

## Description of proposed techniques

This section is concerned of with describing the implemented algorithms MLP, SVM, RF, NB and Voting.

### Multilayer perceptron

**Fig. 1:**
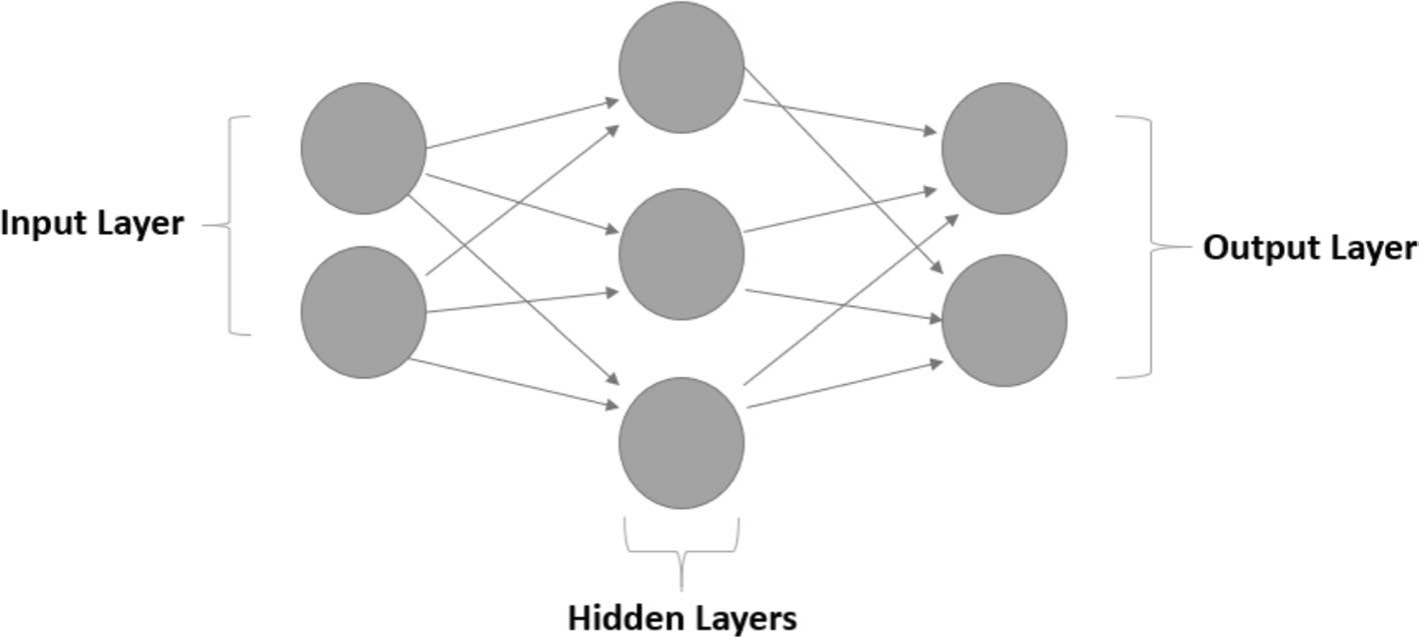
MLP Algorithm flowchart

The MLP algorithm was introduced by M. Minsky and S. Pappert in 1969. This algorithm consists of a neural network that contains multiple layers of nodes. The layers are subdivided into three categories: input layer, hidden layers, and output layer. Furthermore, this algorithm processes data by passing it from the input layer to the hidden layers, and up to the output layer to obtain the classification results [28]. Figure 1 shows the grid configuration of the algorithm, explaining the connections and nodes between the layers.

The input data is fed into the input layer and the extracted data is delivered to the output layer. The hidden layers are layers of nodes between the input and output layers, and there may be one or more of these layers, which perform non- linear transformations on the inputs entered into the network. They are layers of mathematical functions, each designed to produce an output specific to an intended result. The connections between the layers are called weights (W), which are normally defined between 0 and 1. The output value of each neuron is calculated in two subsequent stages as follows. In the first stage, the weighted summation of the input values is calculated using the following Eq. (1):

1 $$\forall l \in \left\{ {1,2, \ldots ,j} \right\},h_{l} = \sum\limits_{i = 1}^{m} {W_{il}^{H} Ii + \beta_{l}^{H} }$$

where Ii is the input variable i, $W_{l}^{H}$. Is the connection weight between i input neuron and the hidden neuron l, m is the total number of inputs and $\beta_{l}^{H}$ is the bias of the *lth* hidden neuron. In the second stage, the output value of each neuron in the hidden layer is calculated based on the weighted summation using an activation function, as in Eq. (2):

2 $$\forall l \in \left\{ {1,2, \ldots ,j} \right\},H_{l} = sigmoid\left( {h_{l} } \right) = \frac{1}{{1 + e^{ - hl} }}$$

The final output is calculated as in Eqs. (3) and (4) [29]:

3 $$\forall k \in \left\{ {1,2, \ldots ,n} \right\},o_{k} = \sum\limits_{i = 1}^{l} {W_{ik}^{o} Hl + \beta_{k}^{o} }$$

4 $$\forall k \in \left\{ {1,2, \ldots ,n} \right\},o_{k} = sigmoid\left( {o_{k} } \right) = \frac{1}{{1 + e^{{ - o_{k} }} }}$$

### Naive Bayes

**Fig. 2:**
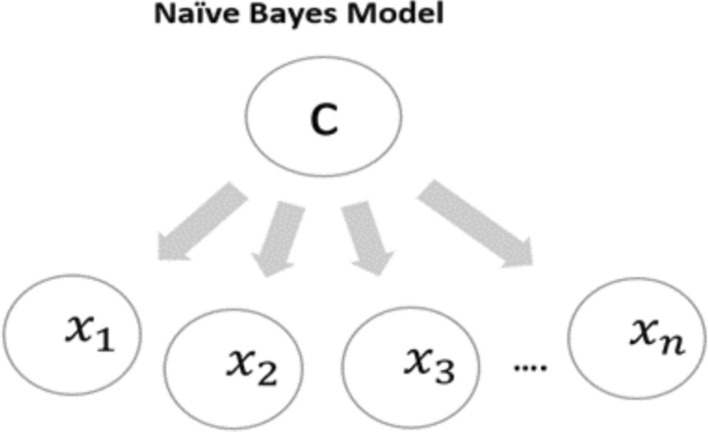
NB Algorithm flowchart

NB is a method that uses knowledge of statistics and probabilities and depends on the implementation of Bayes theory. Figure 2 shows how the probabilistic model provides the probability distribution of an instance over a set of classes. In addition, C is the instance where X1…Xn are the classes, and each probability should be calculated with all classes. This model is the opposite of the deterministic model that only outputs whether or not an instance belongs to positive or negative classes [30].

The mathematical expression for Bayes’ theorem [30] is as follows in Eq. (5):

5 $$P\left( {A\left| B \right.} \right) = \frac{{P\left( {\left. B \right|A} \right) \cdot P\left( A \right)}}{P\left( B \right)}$$

is given in Eq. (5) above. In the NB classifier, all attributes are separated to provide the value of the class variable (depending on independence), as in Eq. (6):

6 $$P\left( {F\left| C \right.} \right) = P\left( {f_{1} ,f_{2} \ldots f_{a} \left| c \right.} \right)_{i}^{n} \pi P\left( {f_{i} \left| c \right.} \right)$$

This algorithm is the easiest and fastest of the Bayesian models [30]. It matches the estimation of the kernel density where it can attain higher levels of accuracy. It works by assuming that all the attributes are independent and affect the results separately [31]. However, this classifier is highly scalpel, requiring several linear parameters for the variables.

### Random forest

**Fig. 3:**
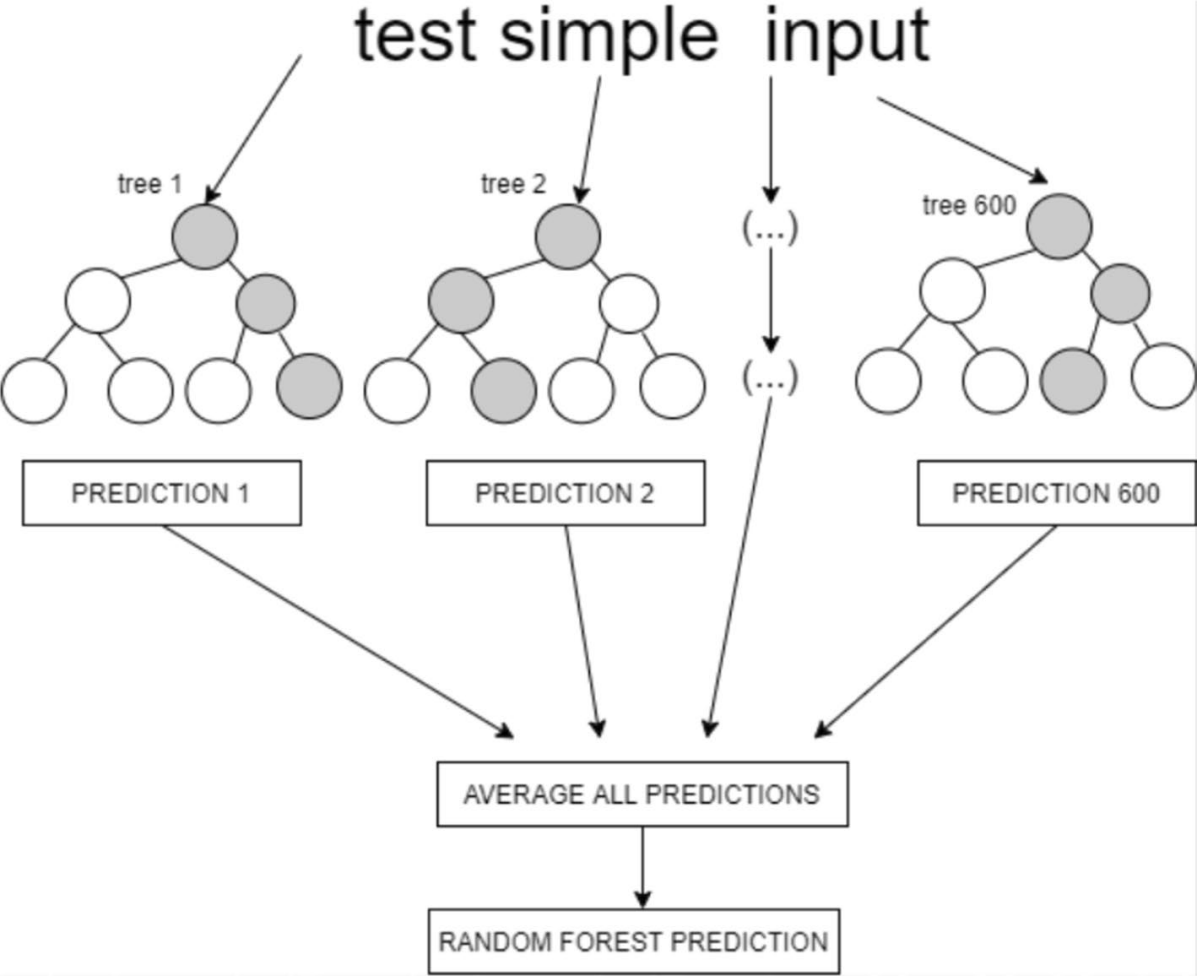
RF Algorithm flowchart

RF is an ensemble classification method. It is designed as a series of classifiers that take a vote on their forecasts in order to classify the data [32]. These classifiers are tree-structured and randomly divide each node between the subsets of the predictors by taking the best-case scenario [33]. In addition, the trees grow using a random set of features. Figure 3 shows the structure of a RF. The trees run in parallel with no interaction between them. During training time, the algorithm immediately constructs several decision trees, picking a random point k from the training set of data points. After that, the first and second steps are repeated by selecting the number of trees, N, that are needed. Ultimately, each of the N-tree trees predicts the value of the output, y, for the data points.

The process is repeated with new data points, then the average value is taken and assigned as the predicted value, y.

### Support vector machines

**Fig. 4:**
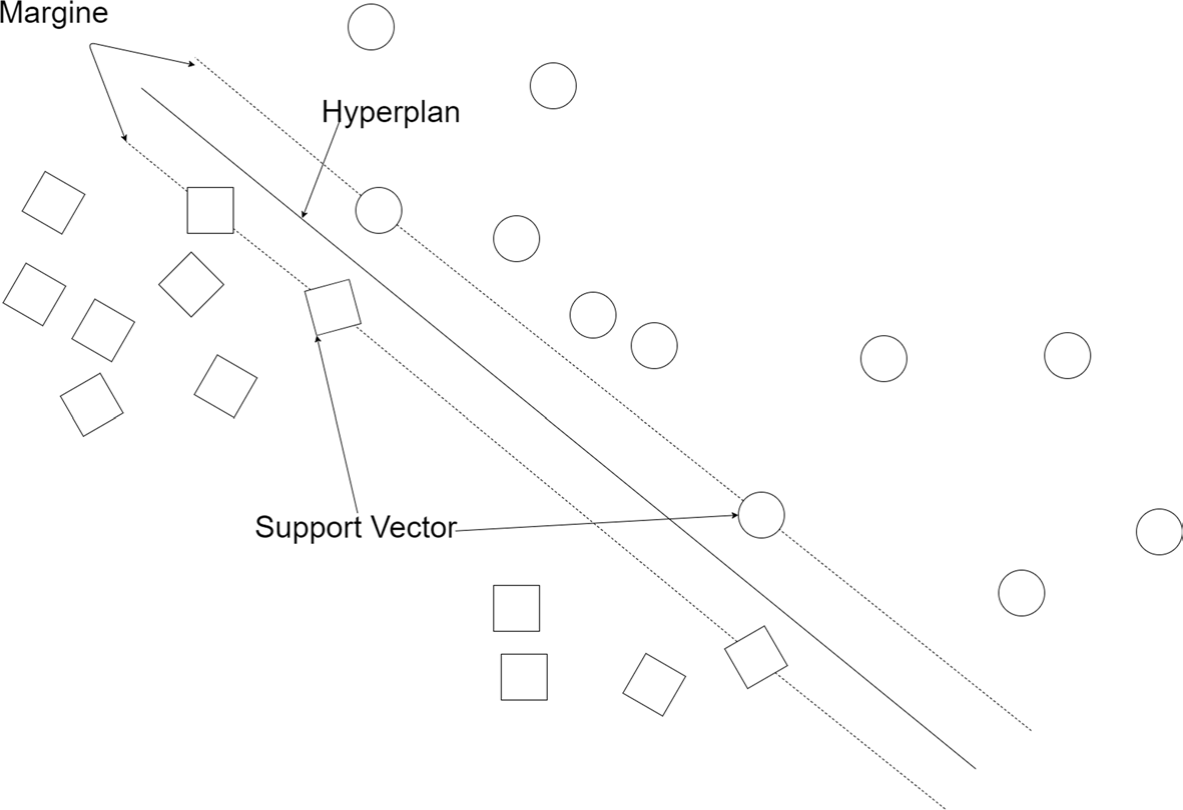
SVM Algorithm flowchart

SVM is a supervised learning algorithm that is mathematically well-founded [32] and is similar to logistic regression. Figure 4 shows how the algorithm works by dividing the sample into two classes by separating the hyper-plane. Furthermore, the few samples at the margin call, support vectors. The distance between the hyper-plane and all training points is called the margin. SVM is recommended to be used in linear model problems. However, one type of SVM, kernel theory, is used to solve nonlinear problems. Linear, polynomial, radial basis function kernels are given in Eqs. (7), (8) and (9) respectively. Linear kernel:

7 $$k\left( {x_{i} ,x{}_{j}} \right) = x_{i}^{T} x_{j}$$

Polynomial kernel:

8 $$k\left( {x_{i} ,x_{j} } \right) = \left( {1 + x_{i}^{T} x_{j} } \right)^{p}$$

Radial Basis Function kernel:

9 $$k\left( {x_{i} ,x_{j} } \right) = e^{{ - \frac{{\left\| {x_{i} - x_{j} } \right\|^{2} }}{{2\delta^{2} }}}}$$

### Voting

**Fig. 5:**
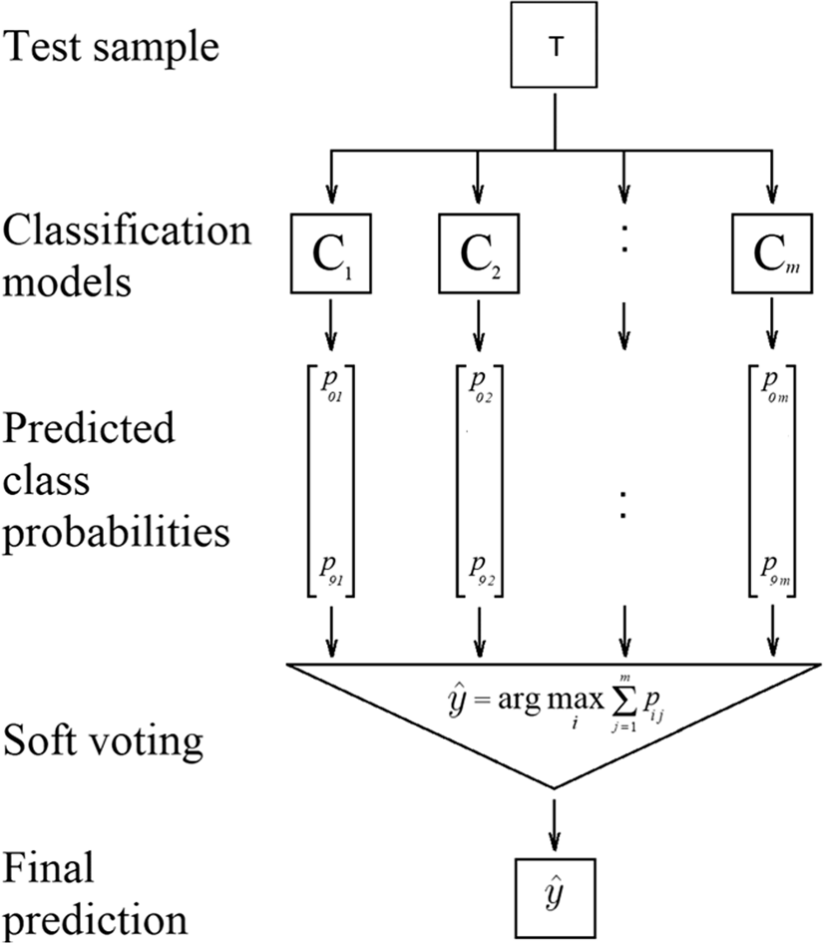
Voting Algorithm flowchart

Ensembling is a method that uses multi-label algorithms together to classify and predict classes. This method is used to optimize the performance obtained from each learning algorithm separately [34]. Furthermore, there are many types of ensemble learning, such as bagging, bootstrapping, stacking and voting [35]. Ensemble voting is used by meta-classifiers to combine ML algorithms by summing the predictions or averaging the predictions made by regression models [36]. Moreover, this classifier is used to aggregate the classes of weak algorithms [37, 38]. Figure 5 shows how this technique sums each classifier with its predicted probabilities to be combined with other classifiers, taking the average for better results.

Finally, the Voting algorithm follows the principle given in Eq. (10) [34],

10 $$\hat{y} = \arg \max i\sum\limits_{j = 1}^{m} {w_{j} p_{ij} }$$

where w_j_ is the weight to be assigned to the j classifier.

For binary classification task with class labels, example i $∈ $0*,* 1

## Methodology

**Fig. 6:**
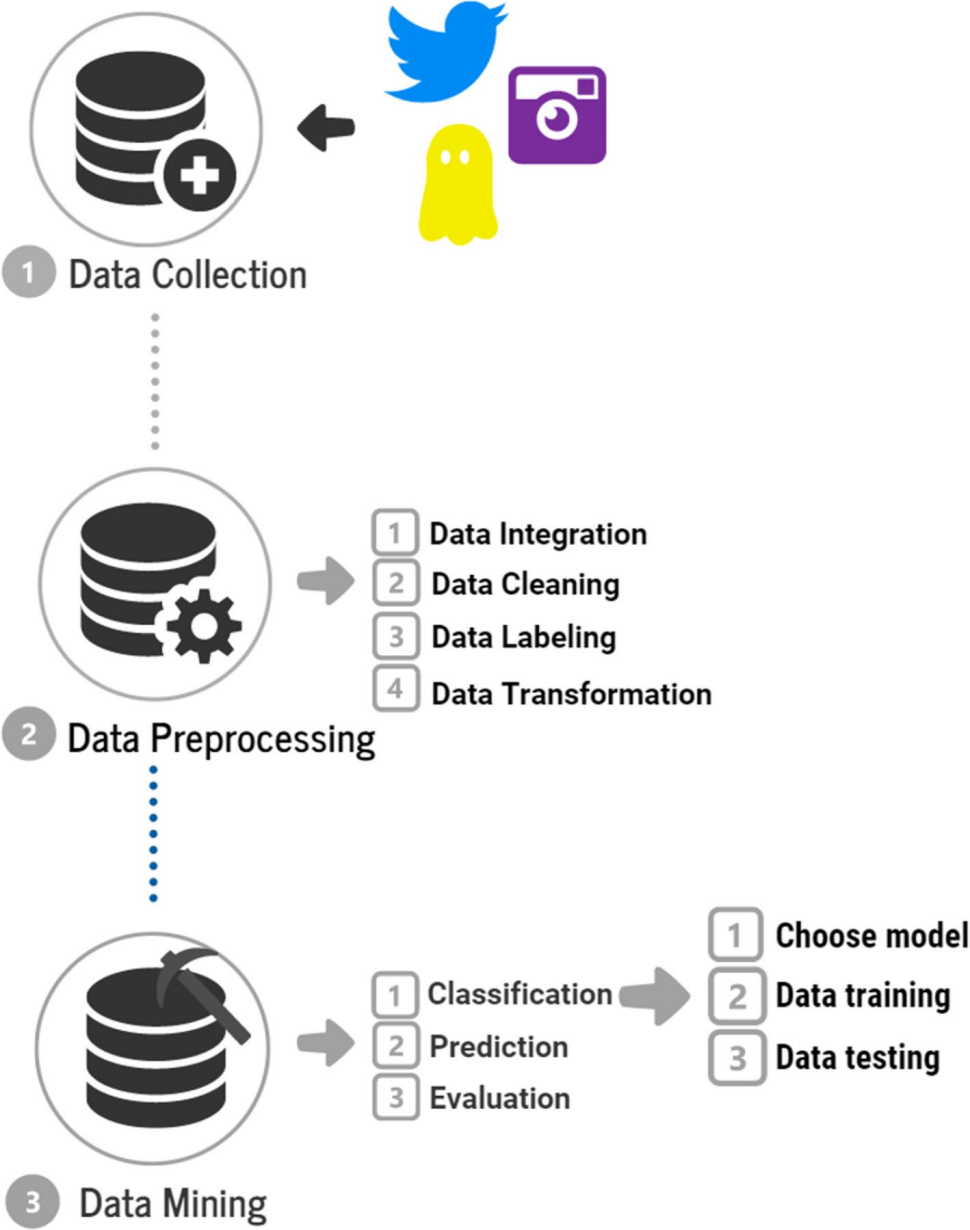
The data mining process framework

This section presents the methods and tools used for data collection and mining from the social networks Instagram, Snapchat, and Twitter. Figure 6 shows the framework for the data mining process, beginning with collecting data from the three platforms, then extracting it, and finishing with the classifying and predicting process.

### Description of dataset

After collecting the data, several features were selected to create a database: gender, text, and class. Table 2 shows the type and utility of each property. The features were chosen according to the content available on each social media platform, in order to compare them.

**Table 2:** Describe each feature

Feature	Type	Description
Gender	Nominal	This column contains a classification of user gender. It contains the two values “F” for a female and “M” for a male. The value is obtained by noting the account name, image displayed, or from the textual data in the contents of the posts
Text	Nominal	This attribute holds values made of strings that have been filtered and converted into vector words. The content of the column is useful for classifying opinions as positive and negative terms are inferred to arrive at the classification decision, whether negative or positive
Class	Nominal	This column is concerned with the results of the instance classification. Class is represented by two nominal values: positive rating and negative rating. The classification result is inferred by analyzing the words and expressions used in the text column

### Experimental setup

In this study, the performance of the implemented ML algorithms is experimentally assessed experimentally, and a comparison is made between five ML algorithms: SVM, RF, NB, MLB, and Voting. The algorithms are applied to the extracted textual data, which is written in Arabic. In addition, the algorithms are tested using the Waikato Environment for Knowledge Analysis (WEKA), applying 10-fold cross-validation and a 70% split as evaluation measures on all imbalance sampled, over-sampled and under-sampled data [39].

**Fig. 7:**
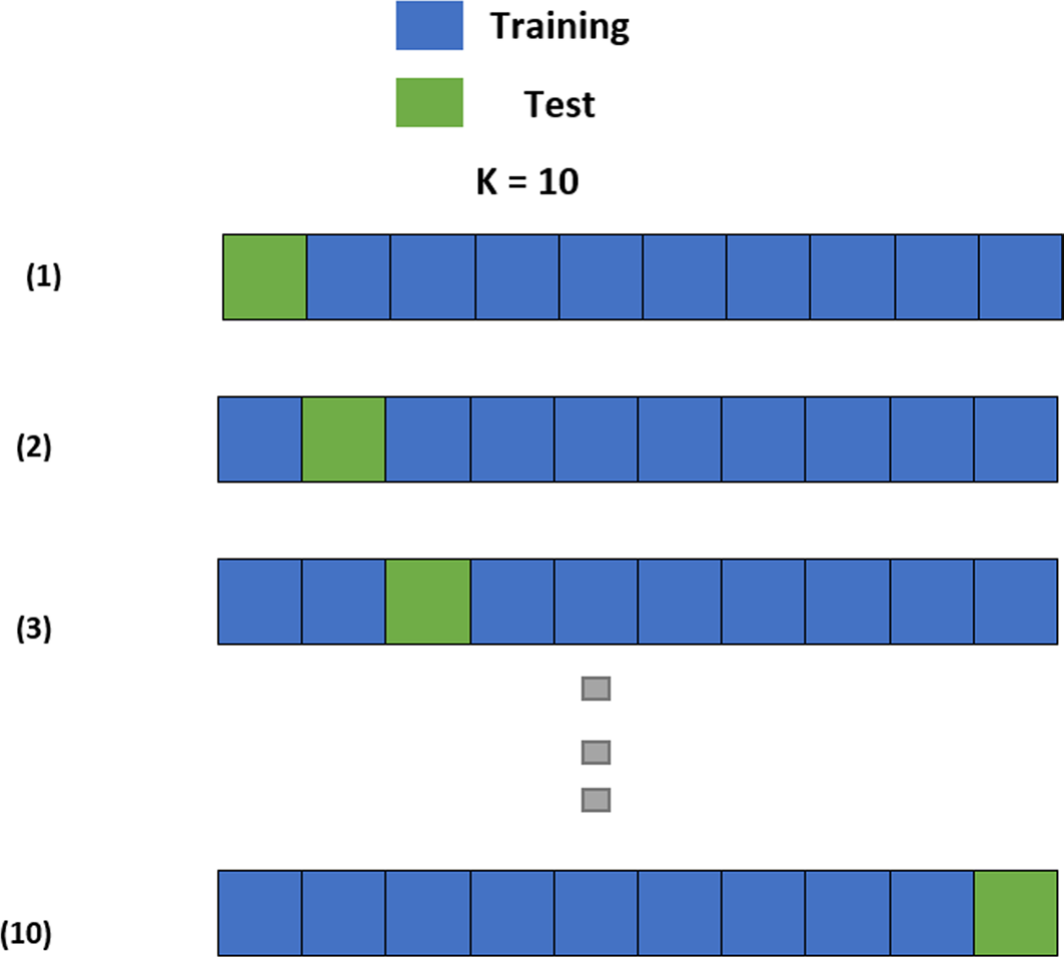
K-fold Cross-Validation

Cross-validation is a method for evaluating predictive models that divide the original sample into a training set and a test set for training and evaluating the model. Figure 7 depicts the data partitioning in ten folds, which implies that the entire data was randomly partitioned into ten parts, nine of which were used to train the model and one used for testing. After that, the process was repeated ten times, with the error being determined each time. The mean of the errors created in each iteration will be the model’s total error [39]. Another way to split the dataset is directly in this research adopted 70% as training dataset and the rest of dataset to testing.

### Data collection

We collected data from different platforms: Instagram, Snapchat and Twitter, from the start of September to the end of October. These platforms were chosen because of their diversity. Through Snapchat, we track the status of tourists in real time, and analyze their feelings by sharing their snaps in Snap Map. Twitter and Instagram were chosen to analyze the comments of tourists and non-tourists by watching the event, and also to compare these different platforms in Sentiment Analysis using ML algorithms. Also, these three applications are the most used in Saudi Arabia, according to what was published by the global media insight [9].

We collected snaps from Snap Map API. The data collection was a real-time process during each trip. The process started with downloading snaps, both pictures and videos, using Python source code. After that, we separated each snap into three layers: textual data, visual content and audio files. The audio files were converted into text using the speech-to-text Python library. The extracted data were recorded in a database file containing snap data from specified map locations in order to build the dataset. On the Twitter platform, we collected the relevant tweets using the Rapid Miner tool to pull out data using keywords. On the Instagram platform, we collected all posts that were either pictures or videos and their comments using hashtags and place tags with the Instaloader tool. Textual data was then manually extracted from these pictures and videos. The keywords used to gather data from Twitter and Instagram were: ‘cruises’, ‘Red Sea’, ‘trip’, ‘prices’, and ‘tourism’.

### Pre-processing

After collecting the data, pre-processing was applied to clean the data of noise. This is the most important factor that can make a difference between a good ML model and a poor one. It attempts to fill in missing values and to smooth out the noise in data. Table 3 contains the sample in the dataset before cleaning and after for each platform.

**Table 3:** Number of datasets before and after pre-processing stage

Platform	Before	After
Instagram	7538	514
Snapchat	1932	284
Twitter	1452	462

**Fig. 8:**
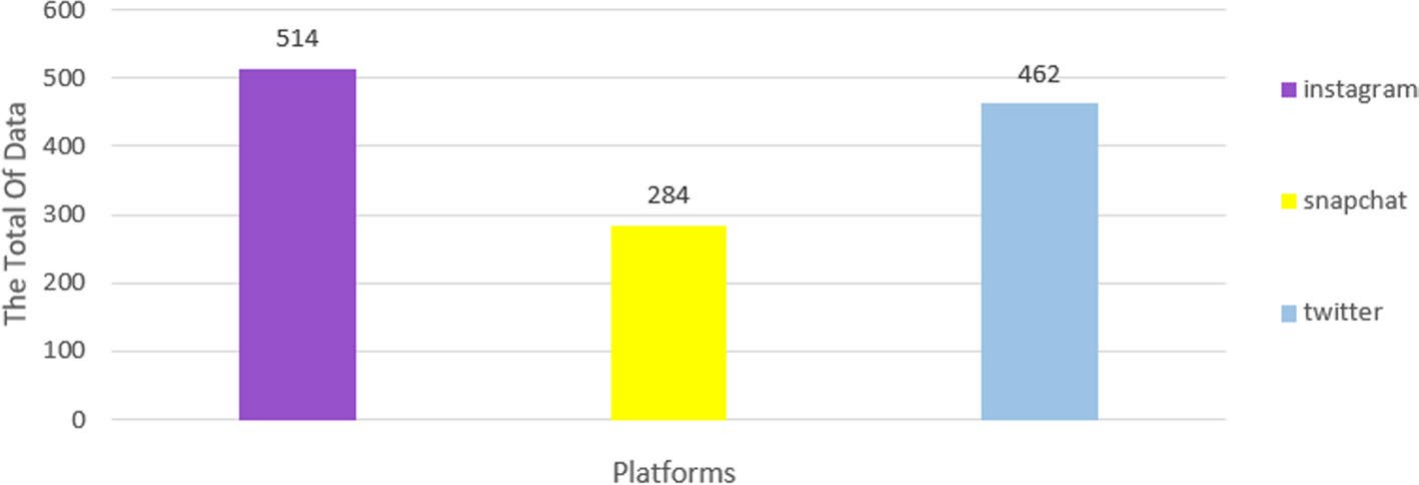
The total of dataset

One of most interesting findings was that interaction on the platforms was highest on Instagram, followed by Twitter, then Snapchat as presented in  Fig. 8.

#### Missing data

- Ignore the attribute: ignore the attribute such as nationality, because it contains several rows with null values
- Fill in a missing value manually: in the case of the categorical feature column, we consider missing data as a new category in itself by replacing the missing values with ‘NA’ or ‘Unknown’ or some other relevant term such as gender column.

#### Noise

Noise is slightly erroneous data observations that do not comply with the trend or distribution of the rest of the data. Though each error may be small, noisy data collectively results in a poor ML model. Noise in data can be minimized or smoothed out by removing the items listed below:

- Arabic diacritics.
- Repeated letters such as “Noooo”.
- Any irrelevant data.
- Numbers such as “123”.
- Elongation.
- Punctuation marks such as $". ! ?-_*[]:;/() "$.
- Focusing on Arabic data and deleting any other language.

### Labeling

**Fig. 9:**
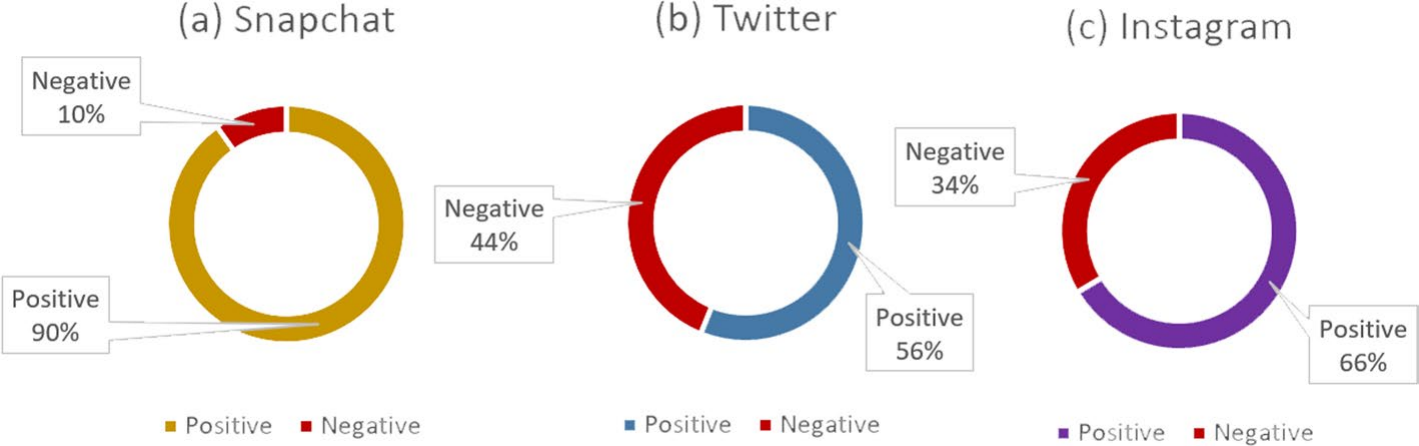
The percentage of positive against negative opinions in all platforms

This section describes the data, called annotation or tagging. This is the process of preparing labeled datasets for ML. Data samples were detected and tagged to establish a foundation for reliable learning patterns. ML systems often require massive amounts of data based on data features that help the model organize the data into patterns that provide an answer. We conducted SA on the sample and labeled it manually as ‘y’, referring to positive sentiment, and ‘x’ referring to negative sentiment. After collecting data from the three platforms, Instagram, Snapchat and Twitter, we configured a separate dataset for each. A total of 10,922 instances were obtained from all platforms and reduced to 1200 after cleaning. In addition, data analysis results show that most of the sample opinions studied were positive about the Saudi Cruise experience. The numbers of positive opinions totaled 342 out of 514 for Instagram users, 256 out of 284 for Snapchat users and 260 out of 462 for Twitter users. This represents 858 positive opinions from the total for all platforms. Figure 9 illustrates the variation of opinions on the three platforms. The results from the data analysis show that the majority of passengers’ opinions were positive about their cruise experience.

### Feature extraction

The n-grams applied by using WEKA refer to a neighboring sequence of n words in a text string, with particular words known to be unigrams (1-g), and n- grams of higher order corresponding to all possible contiguous substrings of length n words that can be constructed from a string. Because of their inherent simplicity, n-grams are a desirable option. An n-gram model can capture more context simply by increasing n.

We hypothesized that the addition of n-gram characteristics would allow a classifier to learn richer representations of the underlying text data, and contribute to a concomitant improvement in the output of classification and useful analysis of sentiment.

### Data transformation to address imbalance in datasets

Imbalances in data are one of the common problems in classification. This phenomenon is increasing in importance since it is faced in natural data domains when the number of samples is unequally distributed between classes by a large ratio. In order to solve such imbalances, a dataset needs to be re-sampled using under- sampling and over-sampling. Under-sampling focuses on the majority class by re- moving samples in order to balance with another class. Conversely, adding samples to the minority class is called over-sampling [40]. Table 4 shows the number of positive (y) and negative (x) opinions in the dataset in terms of imbalance, under- sampling, and over-sampling for all platforms.

**Table 4:** Number of positive and negative sentiment when data re-sampling

Platforms		Number of positive (y)	Number of negative (x)
Instagram	Imbalance	342	172
	Over-sampling	342	342
	Under-sampling	170	170
Snapchat	Imbalance	256	28
	Over-sampling	256	256
	Under-sampling	28	28
Twitter	Imbalance	260	202
	Over-sampling	260	260
	Under-sampling	200	202

### Optimization strategy

#### Multilayer perceptron

**Fig. 10:**
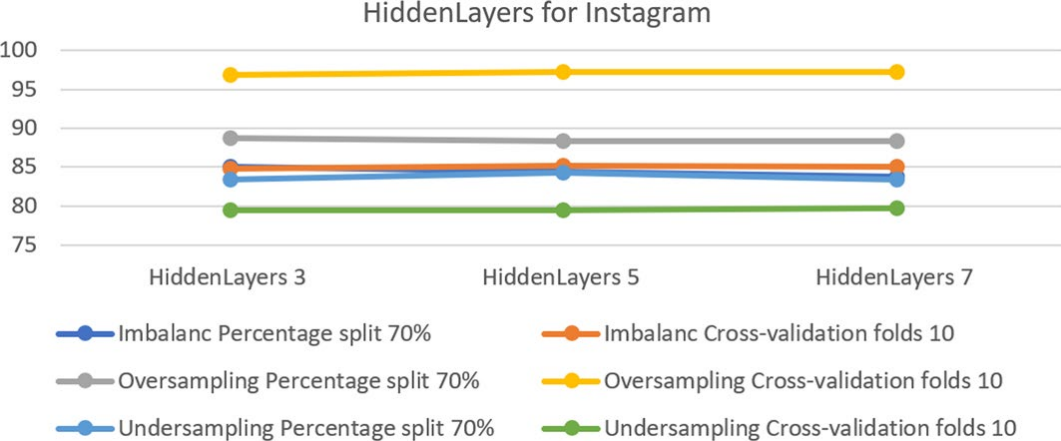
The accuracy of hidden layers for MLP in Instagram

In the MLP model, there is a parameter that allows some changes in the hidden layers when changed to 3, 5 and 7. Moreover, the accuracy changes in some datasets. As shown in  Fig. 10, which compares hidden layers in the Instagram dataset, the effect of increasing the number of layers is to improve the accuracy. It achieved 85.21 in hidden layer 5 in the dataset with imbalance and cross-validation, which was higher than in hidden layer 3. Also in the over-sampled dataset with cross- validation, it achieved 97.22 in hidden layers 5 and 7, the best accuracy for this algorithm. For the under-sampled dataset, meanwhile, with percentage 70% split, the best result was in hidden layer 5, achieving an accuracy of 84.31%.

**Fig. 11:**
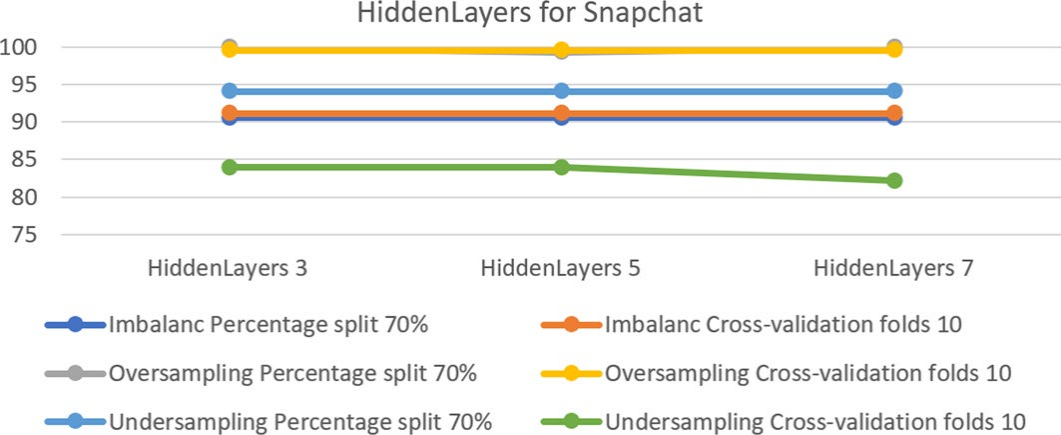
The accuracy of hidden layers for MLP Snapchat

On another platform, Snapchat, there was no change in the imbalance in both tests; all results were equal. However, for the over-sampled dataset hidden layers 3 and 7 gave the best accuracy of 100% with 70% split. In the under-sampled dataset the results were equal except in hidden layer 7, which achieved 82.14% in cross- validation. Figure 11 shows the results obtained from the experiment.

**Fig. 12:**
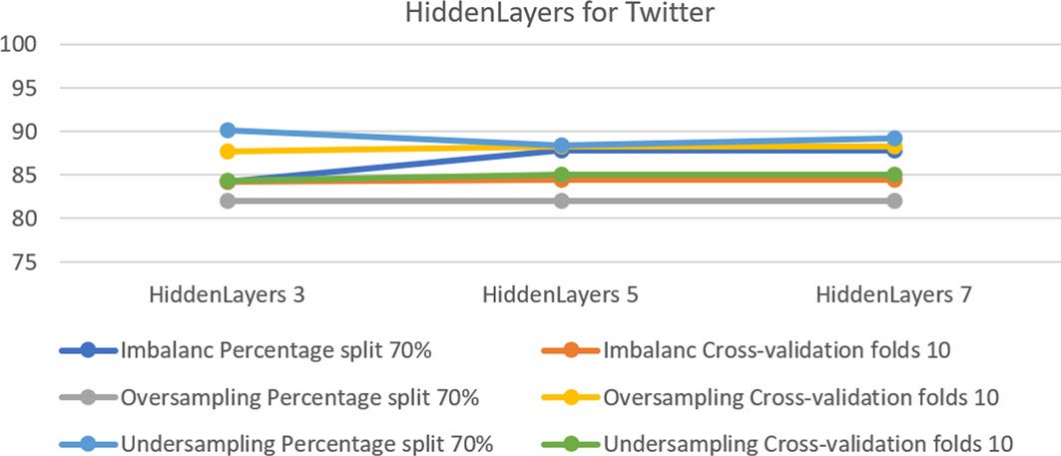
The accuracy of hidden layers for MLP Twitter

The next platform is Twitter. Figure 12 shows that the best results for the dataset with imbalance are 87.77% in both hidden layers 5 and 7 with percentage 70% split. For the over-sampled dataset, the cross-validation test achieved 88.27% in both hidden layers 5 and 7. For the under-sampled dataset, the best accuracy of 90.08% was in hidden layer 3 with a 70% split.

In the MLP model, some parameters did not enable the model to operate properly. These parameters are ‘nominal to binary filter’, ‘normalize attributes’ and ‘normalize numeric class’. Changing the setting from true to false allowed the model function correctly. Table 5 summarizes the optimal parameters for both 10-fold cross-validation and a 70% split.

**Table 5:** Optimal parameter for MLP model

Parameter	Platform	Under-sampling		Over-sampling		Imbalance	
		Fold 10	70%	Fold 10	70%	Fold 10	70%
HiddenLayers	Instagram	7	5	5	3	5	3
	Snapchat	3	3	3	3	3	3
	Twitter	5	3	5	3	5	5

#### Naive Bayes

**Fig. 13:**
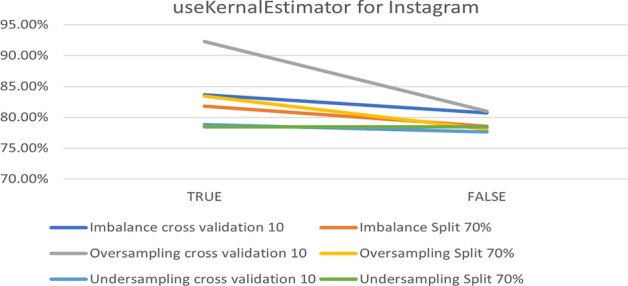
The accuracy of useKernalEstimator for NB in Instagram

In the NB model, the parameter that makes changes is useKernalEstimator, when it is changed from ‘false’ to ‘true’. Moreover, the accuracy changes in some datasets. Figure 13 compares the experimental parameter using the Instagram dataset and shows that changing the value from ‘true’ to ‘false’ leads to 77.64% accuracy in under-sampling with 10-fold cross-validation. It can be illustrated that the best accuracy is related to the default parameters in over-sampling with 10-fold cross- validation.

**Fig. 14:**
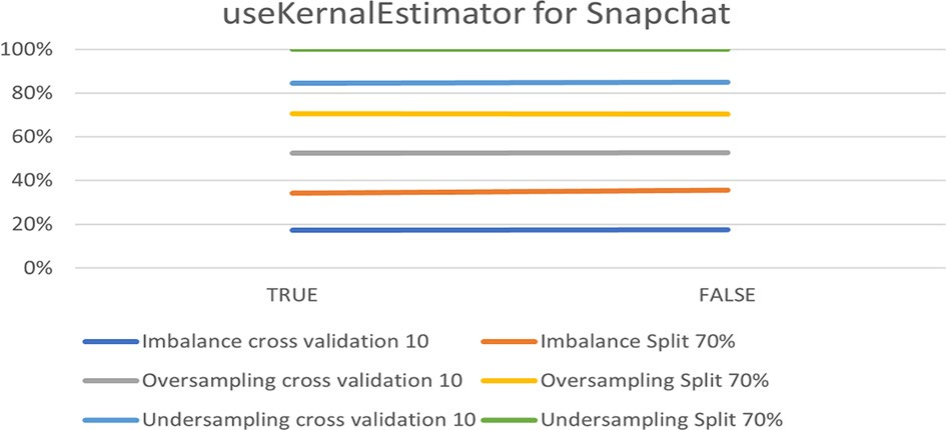
The accuracy of useKernalEstimator for NB in Snapchat

The parameter adjustment was beneficial to under-sampling of the Snapchat platform. However, the default parameters were the best in over-sampling, especially in the 10-fold cross-validation, which achieved 98.04%. Figure 14 shows the results obtained from the experiment.

**Fig. 15:**
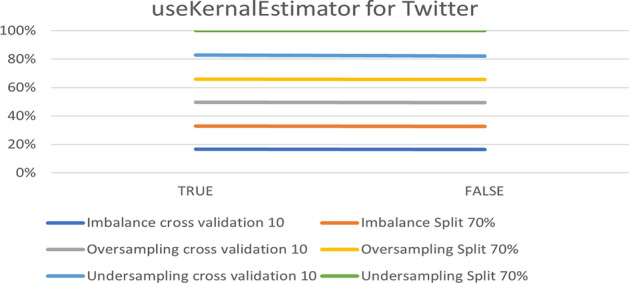
The accuracy of useKernalEstimator for NB in Twitter

The next platform is Twitter. Figure 15 presents an overview of the impact of the parameter on accuracy. The best accuracy is 90.08% for under-sampling with a 70% split.

To summarize the optimal parameters, the default was preserve filters. Such a filter type was normalized through training data, except for the useKernalEstimator parameter. Table 6 summarizes the optimal parameters for both 10-fold cross- validation and a 70% split.

**Table 6:** Optimal parameter for Naive Bayes model

Parameter	Platform	Under-sampling		Over-sampling		Imbalance	
		Fold 10	70%	Fold 10	70%	Fold 10	70%
useKernalEstimator	Instagram	True	True	True	True	True	True
	Snapchat	True	True	True	True	False	True
	Twitter	True	True	True	True	True	True

### Random forest

**Fig. 16:**
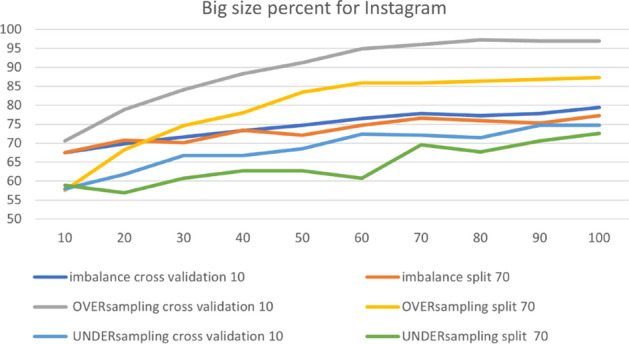
The accuracy of BigSizePercent for RF in Instagram

In the RF model, we changed one parameter, the BigSizePercent, from the de- fault values, with accuracy improving or worsening depending on each option in the dataset. Figure 16 presents the comparison between the parameters for the Instagram dataset. The figure shows that changing the value from 100 to 80 leads to 97.22% accuracy in over-sampling with 10-fold cross-validation. From the chart, we can observe that the best accuracy is for the default parameters in over-sampling with 10-fold cross-validation.

**Fig. 17:**
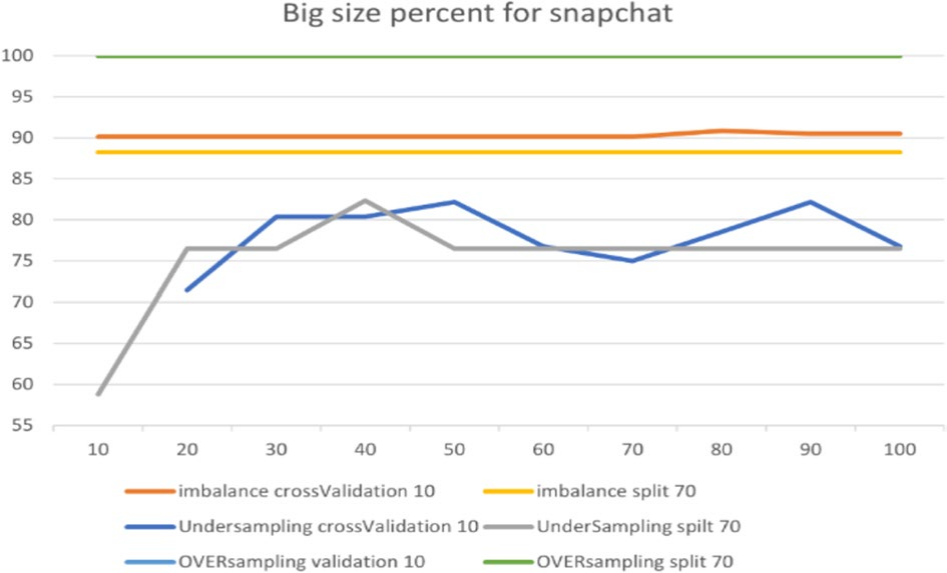
The accuracy of BigSizePercent for RF in Snapchat

The parameter adjustment was useful for imbalance sampling of the Snapchat platform. However, the default parameters performed best in over-sampling, especially with both 10-fold cross-validation and a 70% split, achieving 100%. Figure 17 shows the results obtained from the experiment.

**Fig. 18:**
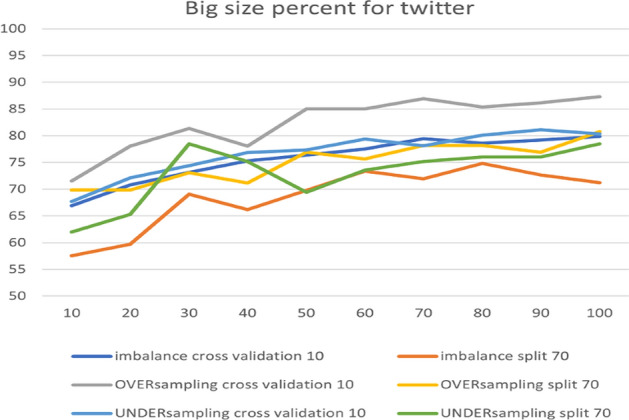
The accuracy of BigSizePercent for RF in Twitter

The next platform is Twitter. Figure 18 presents an overview of the parameter’s impact on accuracy, with the best accuracy being 87.30% for over-sampling with 10-fold cross-validation.

Table 7 summarizes the optimal parameters for both 10-fold cross-validation and 70% split.

**Table 7:** Optimal parameter for BigSizePercent

Parameter	BigSizePercent			
	Platform	Instagram	Snapchat	Twitter
Under-sampling	10-fold	100	90	90
	70%	100	40	100
Over-sampling	10-fold	80	100	100
	70%	100	100	100
Imbalance	10-fold	100	80	100
	70%	100	100	80

### Support vector machine

**Fig. 19:**
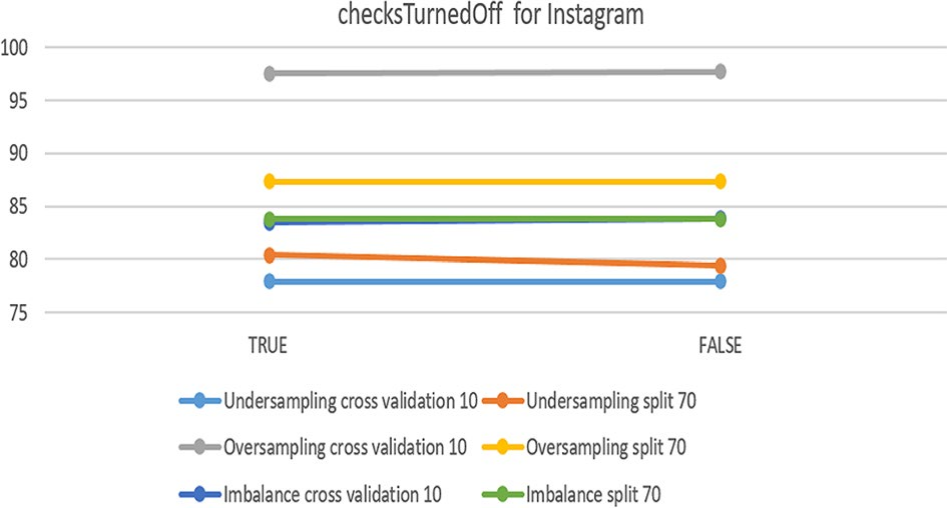
The accuracy of checksTurnedOff for SVM in Instagram

In the SVM model, the only parameter that makes changes is checksTurned Off, when it is changed from ‘false’ to ‘true’. Accuracy changes in some datasets. Figure 19 compares the experimental parameter for the Instagram dataset and shows that changing the value from ‘true’ to ‘false’ leads to 80.39% accuracy in under-sampling with 10-fold cross-validation. From the chart, it can be shown that the best accuracy is related to the default parameters in over-sampling with 10-fold cross-validation.

**Fig. 20:**
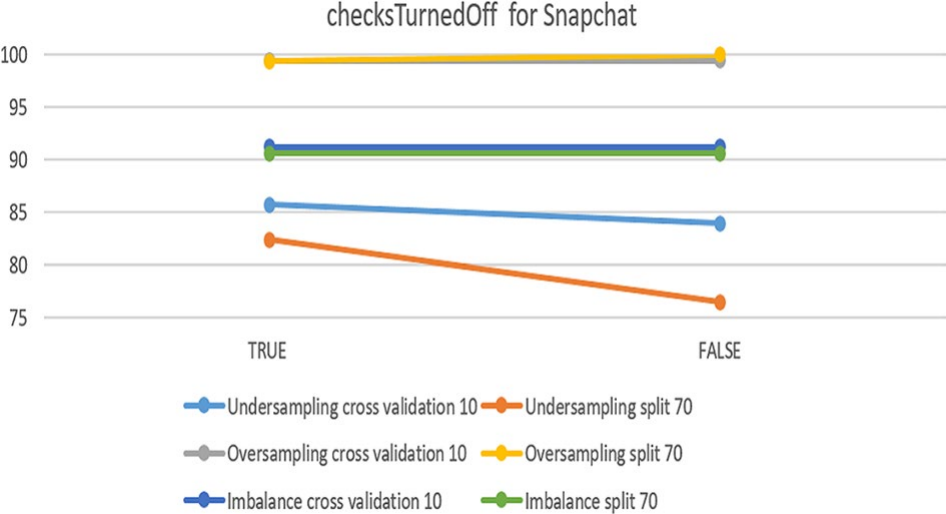
The accuracy of checksTurnedOff for SVM in Snapchat

For the Snapchat platform, parameter change was helpful with under-sampling. However, the default parameters were best in over-sampling, especially 70% split, which achieved 100%. Figure 20 show the results obtained from the experiment.

**Fig. 21:**
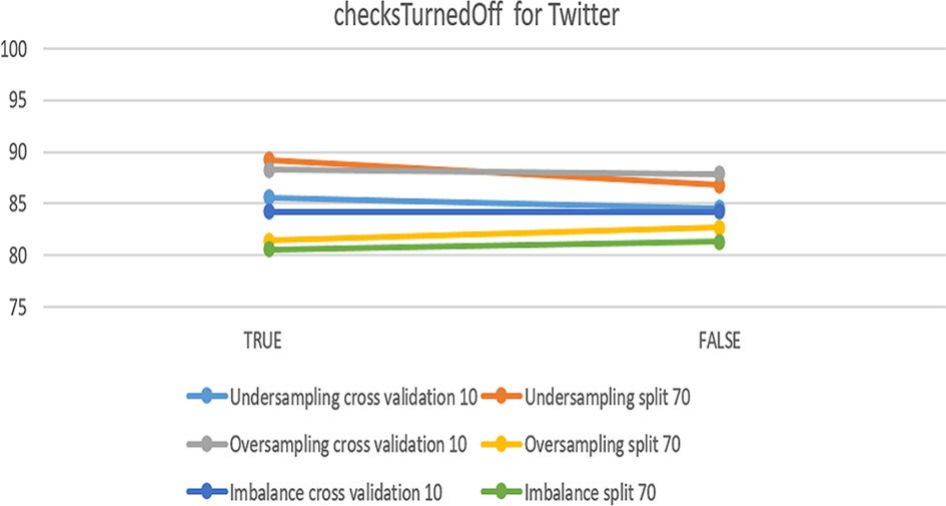
The accuracy of checksTurnedOff for SVM in Twitter

The next platform is Twitter. Figure 21 presents an overview of the impact of the parameter on accuracy. It had no effect on imbalanced data nor on over-sampling with a 70% split, while the best accuracy was 89.26% in the case of under-sampling with a 70% split.

To summarize the optimal parameters, the default was preserved, the filter type was normalized training data, and the kernel was PolyKernel and others, except for checksTurnedOff. Table 8 summarizes the optimal parameters for both 10-fold cross-validation and 70% split.

**Table 8:** Optimal parameter for the SVM model

Parameter	checksTurnedOff			
	Platform	Instagram	Snapchat	Twitter
Under-sampling	10-fold	True	False	True
	70%	True	True	True
Over-sampling	10-fold	False	False	True
	70%	False	False	False
Imbalance	10-fold	False	False	False
	70%	False	False	False

### Voting

**Fig. 22:**
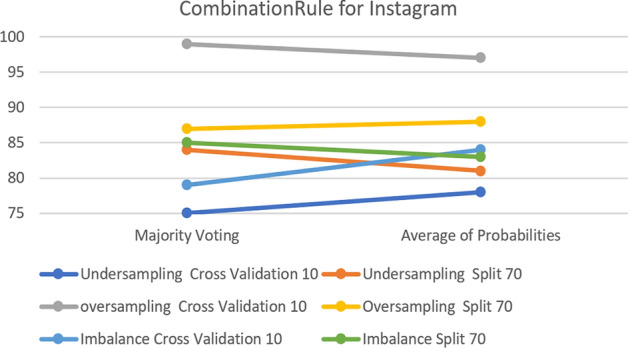
The accuracy of CombinationRule for the Voting Classifier in Instagram

For the Voting classifier, CombinationRule is the only parameter that affects the algorithm accuracy results. Furthermore, the changes are almost negligible, with the maximum accuracy change being almost 3% for all datasets. Figure 22 compares the experimental parameter for the Instagram dataset. The results show that the biggest difference is between Average of Probabilities (AoP) and Majority Voting (MV) in over-sampling, with a 3% change in 10-fold cross-validation, which is the default parameter with the best accuracy from among all the options.

For the Snapchat and Twitter platforms, the results show that keeping the default parameter generates greater classification accuracy in all cases for both platforms.

**Fig. 23:**
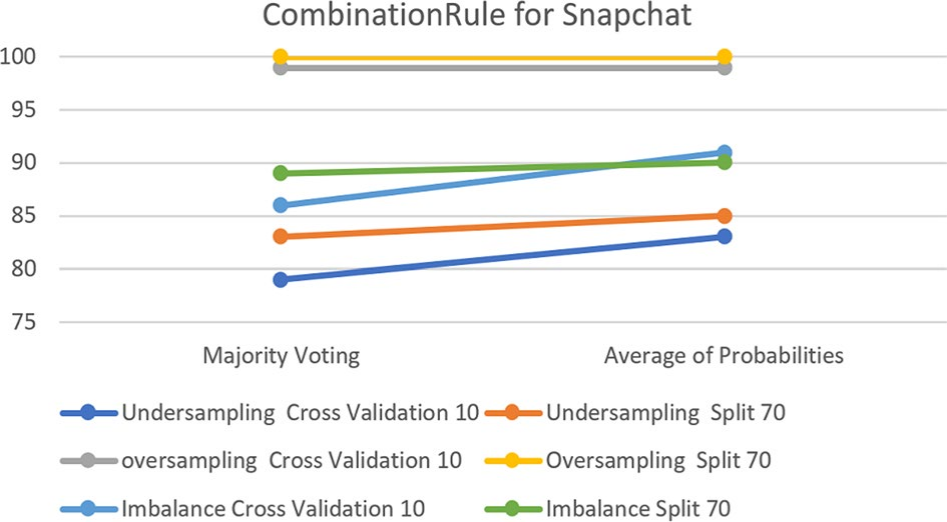
The accuracy of CombinationRule for the Voting Classifier in Snapchat

**Fig. 24:**
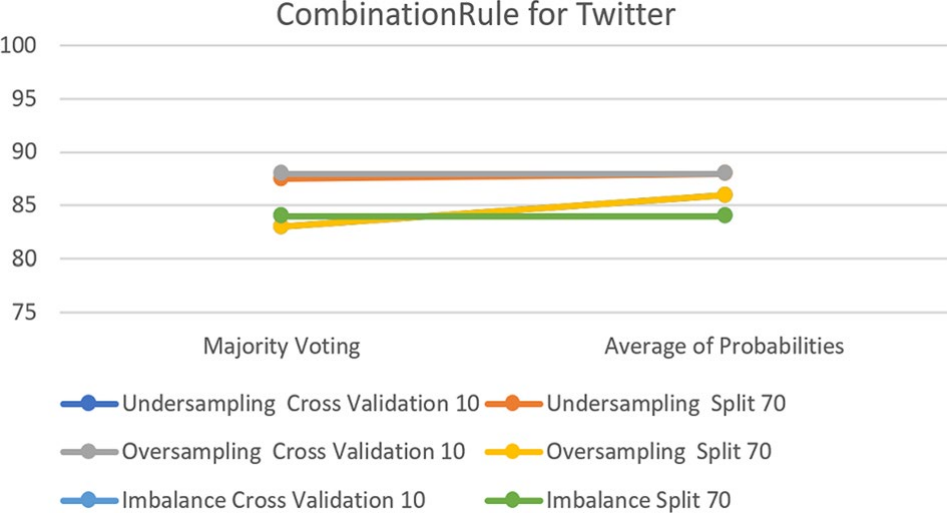
The accuracy of CombinationRule for the Voting Classifier in Twitter

Figure 23 shows that over-sampling has almost the same accuracy before and after sampling, with 100% before and 99% after in Snapchat with the over-sampling cross- validation option. Moreover, it shows that most of the samples have greater accuracy with the parameter set to the default. Finally,  Fig. 24 shows the accuracy change for the Twitter Platform which clarifies the similarly of the impact of changing the parameter on both platforms.

Table 9 summarizes the optimal parameters for both 10-fold cross- validation and 70% split.

**Table 9:** Optimal parameter for the Voting model

Parameter	CombinationRule			
	Platform	Instagram	Snapchat	Twitter
Under-sampling	10-fold	AoP	AoP	AoP
	70%	AoP	AoP	AoP
Over-sampling	10-fold	MV	AoP	AoP
	70%	AoP	AoP	AoP
Imbalance	10-fold	AoP	AoP	AoP
	70%	AoP	AoP	AoP

## Result and discussion

This section presents an overview of the various empirical findings. After applying the algorithms to the datasets, there was a marked difference in terms of their accuracy on each platform. Table 10 shows the results of applying the algorithms on the Instagram platform with split 70% and 10-fold cross-validation test options on imbalanced, over-sampled and under-sampled data. The results show that the SVM algorithm achieved the best accuracy of 97.66%.

**Table 10:** Result of Instagram platform

Instagram							
Algorithm	Metrics	Imbalance		Over-sampling		Under-sampling	
		70%	Fold 10	70%	Fold 10	70%	Fold 10
MLP	Accuracy	84.41%	85.21%	88.29%	97.22%	84.31%	79.41%
	MAE	1.19	0.18	0.14	0.48	0.21	0.23
	Precision	0.85	0.85	0.89	0.97	0.85	0.8
	Recall	0.84	0.85	0.88	0.97	0.84	0.79
	F-Measure	0.84	0.85	0.88	0.97	0.84	0.79
SVM	Accuracy	83.77%	83.85%	87.32%	97.66%	80.39%	77.94%
	MAE	0.16	0.16	0.12	0.02	0.19	0.22
	Precision	0.85	0.84	0.89	0.98	0.81	0.80
	Recall	0.84	0.84	0.87	0.98	0.80	0.87
	F-Measure	0.83	0.83	0.87	0.98	0.80	0.78
RF	Accuracy	77.27%	79.38%	87.32%	97.22%	72.55%	74.71%
	MAE	0.29	0.28	0.28	0.21	0.38	0.37
	Precision	0.81	0.83	0.88	0.97	0.76	0.79
	Recall	0.77	0.79	0.86	0.97	0.72	0.74
	F-Measure	0.73	0.76	0.86	0.97	0.72	0.74
NB	Accuracy	81.82%	83.66%	83.41%	92.25%	87.43%	78.82%
	MAE	0.20	0.19	0.18	0.11	0.22	0.23
	Precision	0.82	0.85	0.86	0.92	0.79	0.79
	Recall	0.82	0.84	0.83	0.92	0.78	0.79
	F-Measure	0.81	0.83	0.83	0.92	0.78	0.79
Voting	Accuracy	85.06%	84.44%	88.29%	97.22%	81.37%	78.82%
	MAE	0.21	0.20	0.17	0.09	0.26	0.26
	Precision	0.86	0.85	0.89	0.97	0.82	0.8
	Recall	0.85	0.84	0.88	0.97	0.81	0.79
	F-Measure	0.84	0.84	0.88	0.97	0.81	0.79

Table 11 shows the results of applying the algorithms on the Snapchat platform with the same settings. The results show that the SVM, MLP, RF and Voting algorithms achieved the best accuracy of 100%.

**Table 11:** Result of Snapchat platform

Snapchat							
Algorithm	Metrics	Imbalance		Over-sampling		Under-sampling	
		70%	Fold 10	70%	Fold 10	70%	Fold 10
MLP	Accuracy	90.59%	91.19%	100%	99.61%	94.12%	83.93%
	MAE	1.10	0.10	0.01	0.01	0.18	0.21
	Precision	0.92	0.90	1.00	1.00	0.95	0.84
	Recall	0.91	0.91	1.00	1.00	0.94	0.84
	F-Measure	0.88	0.89	1.00	1.00	0.94	0.84
SVM	Accuracy	90.59%	91.20%	100%	99.41%	82.35%	85.71%
	MAE	0.09	0.09	0.00	0.01	0.18	0.16
	Precision	0.91	0.90	1.00	0.99	0.87	0.84
	Recall	0.91	0.91	1.00	0.99	0.82	0.84
	F-Measure	0.89	0.89	1.00	0.99	0.82	0.84
RF	Accuracy	88.24%	90.85%	100%	100%	82.35%	82.14%
	MAE	0.12	0.12	0.04	0.03	0.39	0.34
	Precision	0.88	0.92	1.00	1.00	0.87	0.82
	Recall	1.0	0.91	1.00	1.00	0.82	0.82
	F-Measure	0.94	0.87	1.00	1.00	0.82	0.82
NB	Accuracy	89.41%	92.61%	97.40%	98.05%	82.35%	75%
	MAE	0.10	0.09	0.05	0.05	0.25	0.25
	Precision	0.91	0.93	0.98	0.98	0.83	0.76
	Recall	0.89	0.93	0.97	0.98	0.82	0.75
	F-Measure	0.85	0.91	0.97	0.98	0.82	0.75
Voting	Accuracy	90.59%	91.55%	100%	99.41%	85.71%	82.35%
	MAE	0.10	0.10	0.028	0.03	0.25	0.23
	Precision	0.92	0.91	1.00	0.99	0.87	0.86
	Recall	0.91	0.92	1.00	0.99	0.82	0.86
	F-Measure	0.88	0.89	1.00	0.99	0.82	0.86

Table 12 shows the results of applying the algorithms on the Twitter platform with the same settings. The results show that the MLP and NB algorithms achieved the best accuracy of 90.08%.

**Table 12:** Result of Twitter platform

Twitter							
Algorithm	Metrics	Imbalance		Over-sampling		Under-sampling	
		70%	Fold 10	70%	Fold 10	70%	Fold 10
MLP	Accuracy	84.17%	84.19%	82.05%	87.69%	90.08%	84.33%
	MAE	0.20	0.18	0.20	0.14	0.14	0.17
	Precision	0.85	0.84	0.84	0.88	0.91	0.85
	Recall	0.85	0.84	0.84	0.88	0.90	0.84
	F-Measure	0.84	0.84	0.82	0.88	0.90	0.84
SVM	Accuracy	81.30%	84.20%	82.69%	88.27%	89.26%	85.57%
	MAE	0.19	0.16	0.17	0.11	0.11	0.14
	Precision	0.82	0.85	0.84	0.89	0.90	0.87
	Recall	0.81	0.84	0.83	0.88	0.89	0.86
	F-Measure	0.81	0.84	0.83	0.88	0.89	0.86
RF	Accuracy	74.82%	79.87%	80.77%	87.31%	78.51%	81.09%
	MAE	0.82	0.83	0.86	0.89	0.85	0.85
	Precision	0.82	0.83	0.86	0.89	0.85	0.85
	Recall	0.75	0.80	0.81	0.87	0.79	0.81
	F-Measure	0.73	0.79	0.80	0.87	0.77	0.81
NB	Accuracy	84.89%	87.45%	85.26%	88.08%	90.08%	88.31%
	MAE	0.17	0.15	0.15	0.13	0.12	0.14
	Precision	0.85	0.87	0.85	0.88	0.90	0.89
	Recall	0.85	0.87	0.85	0.88	0.90	0.88
	F-Measure	0.85	0.87	0.85	0.88	0.90	0.88
Voting	Accuracy	84.17%	84.89%	84.44%	88.65%	88.43%	86.07%
	MAE	0.22	0.17	0.20	0.17	0.18	0.20
	Precision	0.84	0.85	0.85	0.88	0.89	0.87
	Recall	0.84	0.85	0.84	0.88	0.98	0.86
	F-Measure	0.84	0.85	0.83	0.88	0.88	0.86

Next, the ROC values are presented in Table 13 for the best results.

**Table 13:** ROC values for the best results

	Algorithm ROC		
	Instagram	Snapchat	Twitter
MLP	0.99	1.00	0.97
RF	0.99	1.00	0.94
NB	0.98	1.00	0.96
SVM	0.98	1.00	0.89
Voting	0.97	1.00	0.97

### Further discussions

#### Interaction by women and men

**Fig. 25:**
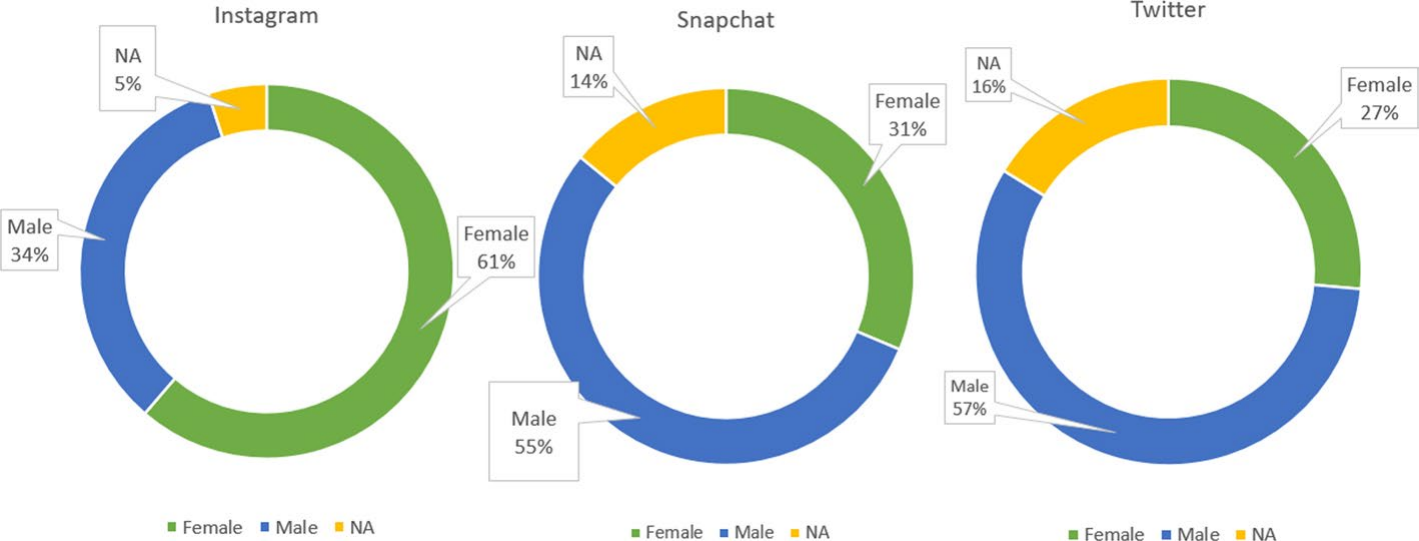
The percentage of gender in all platforms

The research sample was separated based on the users’ gender into females, males and unknown. Each of the three categories were compared to identify the most interactive participants. Figure 25 shows that most Instagram users were females representing over 60% of the sample. However, for the other platforms more than half of the participants were men.

#### Most used words

**Fig. 26:**
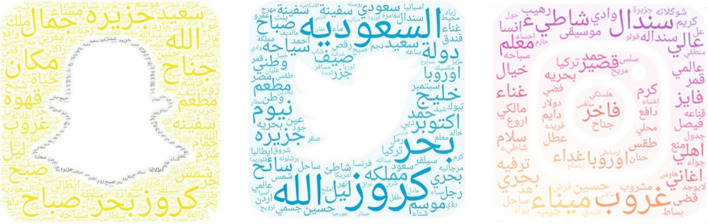
The word clouds of the platforms

In this section, the common words on the three platforms, Instagram, Snapchat and Twitter, are discussed. Figure 26 represents the most used words on Instagram, which were ‘beach’, ‘Sindalah Island’ and ‘sunset’. In addition, for Snapchat the most used words are shown in Fig. 26b; these were sea, cruise and island. Finally, Fig. 26c presents the most popular words on Twitter, such as ‘October’, ‘cruise’ and ‘summer’. In general, most words are positive and the word ‘cruise’ is often mentioned.

## Conclusion and recommendation

In this study, SA was applied to the feelings of passengers and viewers of the Saudi Cruise, the first cruise in the Kingdom of Saudi Arabia. The sample was collected from three social media platforms, Snapchat, Twitter and Instagram. Separate datasets for each platform were created, and we obtained 10,922 instances, which were reduced to 1200 after cleaning. The results showed that most opinions, 80%, were positive across all three platforms. Furthermore, the ML algorithms, MLP, SVM, NB, RF and Voting, were applied to each dataset in order to classify and predict the opinions of passengers and viewers. The results show that the algorithms, RF, SVM and Voting, are the best when applied to the Snapchat platform, while the RF, SVM and Voting algorithms are best for Instagram, and NB and MLP for Twitter.

In addition, the dataset analysis showed that the most used words were ‘cruise’, ‘Saudi’ and ‘Allah’. These words may be explained by the fact that’ cruise’ relates to the kind of trip,’ Saudi’ relates to the location of the trip. As for the word ‘Allah’, it is an Arabic word that means ‘God’. The word ‘Allah’ stands for the surprising, the beautiful, the amazing, etc. Also, 80% of those who shared their experience were men. The difference between the gender of passengers who published their experiences is attributed to different circumstances that led to a decrease in women’s participation on social media platforms, including the conservative culture of Saudi society.

This study responds to this need. The starting point for the study was to monitor the interactions between people on social media during the first cruises in Saudi Arabia during the COVID-19 pandemic as a unique experiment. Hence, the SA perspective should be the first step in any attempt to examine public opinion on any development or change. Thus, ML models were designed and developed to improve the process parameters in predicting feelings in the future that will help the decision-maker. Also, performance analysis enhanced models so that they achieved 100% accuracy. In addition, the social media platforms were compared in order to detect feelings, which is innovative.

This study has potential limitations. The data considered were from three popular social media platforms, Instagram, Twitter and Snapchat. Also, the period for data collection was long compared to the amount of data in the dataset. Because of Covid-19 and social distancing, the number of passengers on trips was reduced. Furthermore, there were places where taking pictures or videos was not allowed in order to protect the privacy of passengers.

A natural progression from this work is to compare more algorithms. These findings provide the following insights for future research: data in different formats, such as visual and audio data, could be analyzed; further research might explore more platforms for data comparison; development of a hybrid algorithm from the algorithms used in this study is recommended. Ultimately, the study could be applied to a wider area of entertainment in Saudi Arabia. And it is recommended that the scope of the study be expanded to other regions and countries by adding other data sets, to include demographics and not be limited to Saudi Arabia.

## Data Availability

The datasets generated during and/or analysed during the current study are available from the corresponding author on reasonable request.
